# A Rare Case of Cow’s Milk Protein Allergy in a 12‐Hour Old Neonate With Rectal Bleeding

**DOI:** 10.1155/crpe/5574716

**Published:** 2026-05-28

**Authors:** Isona Kakuchi, Alexa Giroud Rivier, Estelle Dubruc, Céline J. Fischer Fumeaux, Sébastien Joye

**Affiliations:** ^1^ Department Mother-Woman-Child, Clinic of Neonatology, Lausanne University Hospital and University of Lausanne, Lausanne, Switzerland, chuv.ch; ^2^ Department Woman-Mother-Child, Pediatric Gastroenterology, Lausanne University Hospital and University of Lausanne, Lausanne, Switzerland, chuv.ch; ^3^ Institute of Pathology, Lausanne University Hospital and University of Lausanne, Lausanne, Switzerland, klinikum-fuerth.de

## Abstract

Hematochezia presenting in the neonatal period requires a thorough exploration of life‐threatening and benign pathologies, including food protein–induced allergic proctocolitis (FPIES). This non‐IgE–mediated food allergy is most commonly associated with cow’s milk protein (CMP). It classically presents in the first weeks to months of life, following the introduction of a cow’s milk–based formula or breast milk where the maternal diet includes cow’s milk. We present a rare case of extremely early onset of CMP‐induced proctocolitis, in a neonate presenting at 12 h of life with hematochezia. How does a food allergy develop at such a young age? We explore the current theories explaining this phenomenon.

## 1. Case Report

A term male neonate was born at 39 weeks and 4 days’ gestation, weighing 4200 g, by spontaneous vaginal delivery, from a gravida 4, para 1 mother. Family history was positive for cow’s milk protein allergy (CMPA) in a first cousin from the maternal side. The pregnancy was unremarkable, apart from a mild maternal viral infection 3 weeks prior to the delivery. The neonate, born in meconium‐stained amniotic fluid, required ventilatory assistance by bag valve mask for 1 minute before establishing spontaneous breathing. The Apgar scores were 3 and 9 at 1 and 5 minutes, respectively. The venous cord pH was 7.4. Intermittent grunting since birth resolved within the first hour of life. The patient received oral vitamin K and was transferred to the maternity ward with his mother.

**FIGURE 1:**
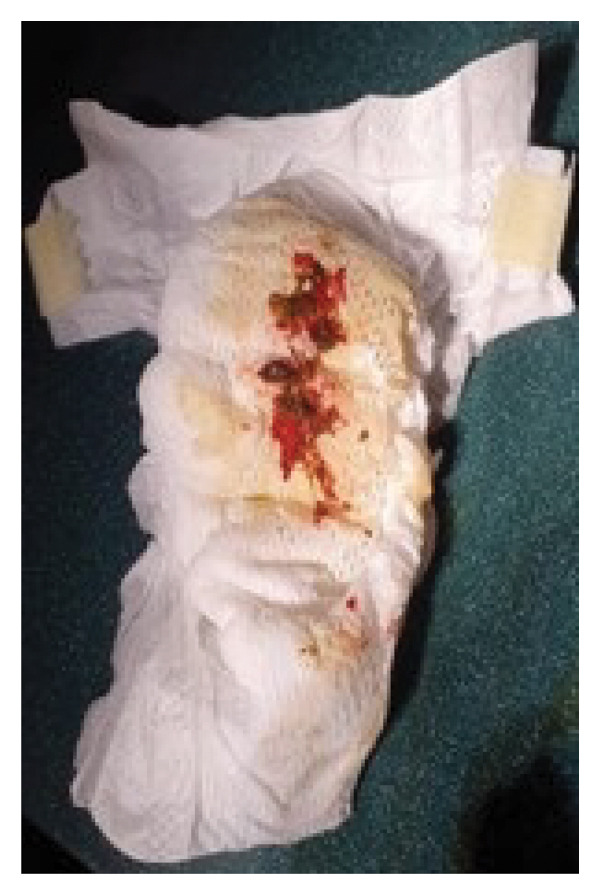
Stool with fresh blood was repeatedly seen from 12 h of life.

At 12 hours of life, the neonate presented an episode of hematochezia (Figure 1). Since birth, he had been regularly breast fed with no vomiting and normal passage of meconium as confirmed by the presence of meconium‐stained amniotic fluid. Vital signs were normal. The physical examination excluded perianal trauma but identified pallor and diffuse abdominal discomfort. The neonate was admitted to the neonatal care intensive unit. A nasogastric tube was inserted for regular gastric aspiration.

**FIGURE 2:**
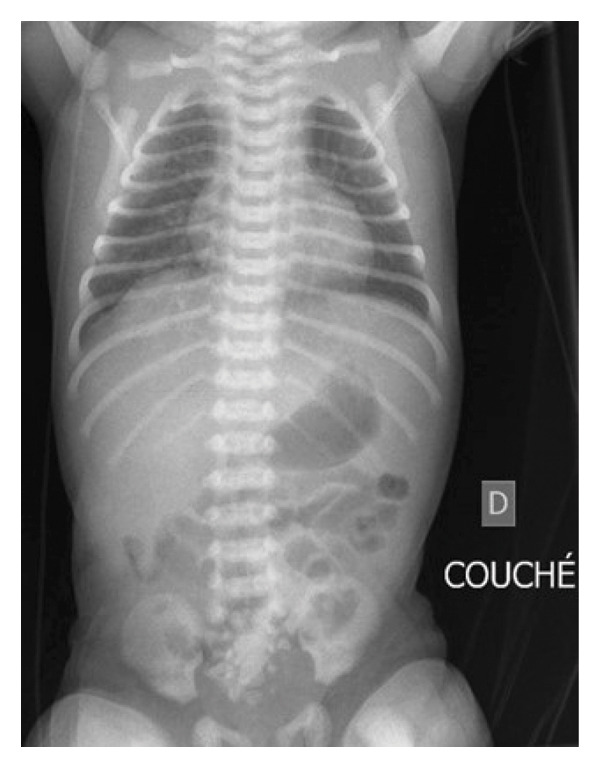
Abdominal X‐ray: The abdominal X‐ray did not show intestinal dilatation, pneumatosis, or free air.

The initial workup with an abdominal X‐ray (Figure 2) and ultrasound with mesenteric Doppler flow assessment enabled rapid exclusion of volvulus, intestinal malrotation, and intussusception. In addition, there were no risk factors nor signs of necrotizing enterocolitis. The full blood count revealed an anemia with a hemoglobin of 113 g/L, indicating a packed red cell transfusion to normalize the hemoglobin to 139 g/L. Conversely, the white blood cell (WBC) and platelet counts, as well as clotting factors, were normal. A negative Apt test ruled out presence of maternal blood in the infant’s stool. Despite a lack of neonatal sepsis risk factors and absence of inflammatory markers (WBC: 14.8 G/L and CRP: 2 mg/L), blood and stool cultures were sampled for cultures. Viral infection was also considered, but urinary *Cytomegalovirus* and viral stool polymerase chain reaction tests were negative.

The patient remained monitored and received a glucose infusion and empiric antibiotics of amoxicillin and gentamycin for a differential diagnosis of sepsis, given the clinical presentation and the potential risk of translocation. Enteral feeding remained suspended. He continued to present episodes of hematochezia. Nevertheless, the rest of the clinical picture was stable, the abdominal examination normalized, and anemia did not recur posttransfusion.

**FIGURE 3:**
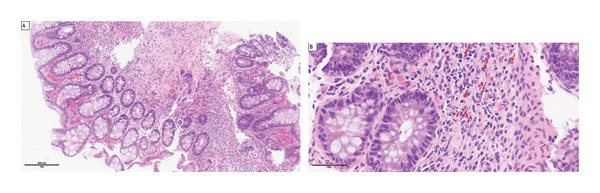
Histology (hematoxylin and eosin [HE] staining) of rectosigmoidal biopsies at low (A) and higher magnification (B) with eosinophilic infiltration of the mucosa. Arrows show eosinophils.

After a multidisciplinary discussion with the pediatric surgeons and gastroenterologists, rarer diagnoses were considered due to lack of clinical improvement, including an arteriovenous malformation originating in the gastrointestinal (GI) tract and Meckel’s diverticulum. However, the clinical and biological evolution did not speak in favor of a severe hemorrhagic event. An allergic origin such as CMPA, though uncommon at this age, was raised as a plausible diagnosis. A rectosigmoidoscopy with biopsy was judged as the next appropriate investigation given this hypothesis, to avoid potentially unnecessary more invasive procedures, and was performed on day of life (DOL) 4. The macroscopic findings revealed mucosal inflammation and friability. Histological analysis showed numerous eosinophils (30 eosinophils per high power field [x400]) in the lamina propria of the mucosa. The mucosa lacked architectural changes (Figure 3). These results suggested CMPA and led to a trial of dietary exclusion by switching from maternal breast milk and regular formula to extensively hydrolyzed formula on DOL 4. Clinical symptoms improved, with resolution of hematochezia and steady increase in growth parameters.

A bacterial origin was excluded given lack of improvement under antibiotic therapy, the rectosigmoidoscopy results, and nonsignificant blood and stool culture results. Antibiotics initiated on DOL 1 were therefore stopped on DOL 5.

Evolution was favorable with full enteral feeding achieved on DOL 9. Following strict maternal dietary exclusion of cow’s milk protein (CMP) for 10 days, breastfeeding was recommenced successfully on DOL 14. Hospital discharge was possible on DOL 17.

Outpatient follow‐up at 6 weeks was encouraging, with complete resolution of hematochezia and a positive growth curve since reintroduction of breastfeeding with strict cow’s milk eviction. Abdominal examination was normal. At 2 months of age, dairy products were reintroduced to the maternal diet and breastfeeding continued with no adverse effects. At 4 months of age, good height–weight growth was observed along with good tolerance of solid foods. Introduction of CMP solid products was therefore attempted at 6 months of age with no issues. Furthermore, breastfeeding was stopped at 7 months with transition to standard cow’s milk formula and well tolerated. At 12 months of age, cow’s milk was introduced successfully. Currently, at 30 months of age, height–weight growth continues to excel and there are no atopic symptoms.

## 2. Discussion

This patient presented with hematochezia 12 hours after birth; complementary investigations and evolution revealed the underlying cause as FPIAP due to CMPA. This is one of the earliest onsets of this disease reported to our knowledge.

In neonatology, the incidence of upper GI bleeding is around 1.2%, and it is 0.3% for lower GI bleeding [1]. However, distinguishing between upper GI and lower GI hemorrhage can be difficult in this age group. Transit is fast, meaning swallowed fresh blood can be directly excreted in stool with no time for digestion. The causes of neonatal rectal bleeding can thus be extremely varied, and a wide diagnostic differential must initially be considered (Table 1) [2].

**TABLE 1:** Diagnostic differential of hematochezia in neonatal patients.

Serious pathology	Benign pathology
*Hematological pathologies*	*Ingestion of maternal blood (intrapartum or from bleeding nipples)* *Anal and/or rectal fissure* *CMPA*
‐Coagulopathy	
‐Hemorrhagic disease of the newborn (vitamin K deficiency)	
‐Hemophilia	
‐Maternal medications (anticoagulant, thiazides, phenobarbital)	
‐Thrombocytopenia	
‐Platelet alloimmunization	
‐TORCH infection	
*Gastrointestinal anatomical anomalies*	
‐Malrotation with volvulus	
‐Intussusception	
‐Meckel’s diverticulum	
‐Polyp	
‐Vascular malformation	
‐Arteriovenous malformation	
‐Hemangioma	
*Infection*	
‐Bacterial gastroenteritis	
‐Viral gastroenteritis: TORCH, rotavirus, adenovirus	
*Gut hypoperfusion*	
‐Necrotizing enterocolitis	
‐Hirschsprung enterocolitis	
‐Septic shock with disseminated intravascular coagulopathy	
‐Vasoactive drugs	
‐Congenital heart disease	

Abbreviations: CMPA, cow’s milk protein allergy; TORCH, Toxoplasma, Other, Rubella, Cytomegalovirus, and Herpes simplex.

Firstly, one must examine for life‐threatening conditions, which could lead to shock. These include hematological abnormalities, intestinal malrotation with volvulus, intussusception, sepsis, and necrotizing enterocolitis. Significant rectal bleeding in a neonate requires, as illustrated in this case, immediate hospitalization, hemodynamic stabilization, and initiation of empiric antibiotic therapy. Initial investigations must include FBC, clotting, blood culture, stool bacterial and viral cultures, and imaging in the form of abdominal X‐ray and ultrasound.

Secondly, benign causes such as ingestion of exogenous blood and anal fissure must not be forgotten. Examination of the mother in breast‐fed neonates, Apt testing of stool, and close examination of the neonate are essential.

Thirdly, rarer causes such as arteriovenous malformation and CMPA must be considered if these initial investigations are unfruitful. If the patient is stable, as was the case in our patient, rare benign causes should be explored as priority to avoid unnecessary, invasive, harmful, and costly procedures. A rectosigmoidoscopy can be performed rapidly and demonstrate signs compatible with food allergy, as observed in our patient.

CMPA is the most common childhood food allergy affecting around 2%–3% of infants with a peak in the first year of life [3, 4]. The underlying adverse immunological reaction can be either IgE, non‐IgE, or mixed IgE and non‐IgE–mediated. The most common type of CMPA is FPIAP, a non‐IgE–mediated allergy, with a prevalence of 1.5%–2% [3, 5]. Infants are sensitized to CMP through repeated postnatal exposure to breast milk or formula milk. Subsequent ingestion of CMP leads to a significant immune response including release of tumor necrotizing factor alpha (TNF‐alpha), resulting in the hallmark symptom of rectal bleeding in an otherwise healthy infant. Severe anemia and failure to thrive are rare. Symptom onset is typically between 2 and 6 weeks of age, but extremely rarely during the first hours of life, as in our patient.

The diagnosis of FPIAP is clinical, with symptom resolution after a period of allergen eviction of 2–4 weeks, then reappearance upon allergen reintroduction. The latter criteria of reappearance of symptoms upon an oral food challenge isunnecessary if the initial clinical picture is convincing and/or the symptoms are severe [3]. In our specific case, an oral food challenge was not performed due to persistent bloody stools and the early timing of the diagnosis after the rectosigmoidoscopy. We acknowledge that rectosigmoidoscopy and histology are not routinely recommended for diagnosis as findings are nonspecific [4]. Nevertheless, they can support a diagnosis of FPIAP in complex cases such as our patient and can be performed easily in the first months of life without general anesthesia. Indeed, mucosal atrophy and eosinophilic infiltration are nonspecific but still typically seen in FPIAP. These findings, interpreted together with the clinical context, can therefore help to exclude other pathologies, avoid more invasive procedures such as an explorative laparoscopy, and accelerate management. On the other hand, skin prick testing and specific IgE titers are not useful for diagnosis of this non‐IgE–mediated allergy.

Treatment is based on temporary elimination of the allergen from the diet. Infants should avoid direct CM consumption and milk containing CMP. For exclusively breastfed infants, mothers should eliminate CM from their diet for 2–4 weeks and may also need to avoid egg and soy if there is no response. If the infant still remains symptomatic, feeds should be switched to an extensively hydrolyzed formula. An amino acid–based formula may be necessary for severe cases. For formula‐fed infants, feeds should be switched to an extensively hydrolyzed formula, then amino acid–based formula depending on response. For infants receiving both formula and breast milk, if symptom relief is achieved upon switching formula, breastfeeding with maternal CM ingestion can be continued in parallel. For all infants, symptoms are expected to improve after 2–4 weeks of CMP exclusion. Subsequent allergen reintroduction, excluding infants presenting with severe symptoms including anaphylaxis, is required for confirmation of the diagnosis and to avoid overdiagnosis. The gold standard is by a double‐blind placebo‐controlled food challenge; however, an open oral food challenge is also acceptable. Reintroduction of direct CM consumption and CM‐based formula is advised after 6 months or at the age of 9–12 months [4]. Prognosis is favorable with over half of infants presenting symptom resolution by 12 months of age and close to 90% by 3 years of age [6].

Our case raises an interesting question regarding this proposed pathophysiology. How then, does a neonate who has had exposure to only a small volume of colostrum since birth elicit a significant allergic response at a mere 12 hours of age? Järvinen et al. hypothesize the process of early sensitization to CMP via breast milk [7]. Mothers of exclusively breastfed infants known to have CMPA followed a CM‐free diet until symptom resolution. Following reintroduction, 94% of infants presented with dermatological and digestive symptoms compatible with CMPA. Feiterna‐Sperling et al. report a neonate at 1 hour of life presenting with hematochezia due to CMPA, and Alabsi reports symptomatology in a neonate even prior to postnatal enteral feeding, suggesting sensitization can occur even earlier, in utero [8, 9]. Certainly in our case, presenting as early as 12 hours of life, in utero sensitization could be the underlying cause.

For intrauterine sensitization to be possible, firstly the fetus must have a relatively mature immune system allowing for sensitization at an early stage. Hopp et al. suggest development of the immune system from the beginning of the first trimester, marked by presence of macrophages and tissue mast cells [10]. By 20‐week gestation, neutrophils and eosinophils are also present and the fetus is primed for mounting an immune response. Secondly, the fetus must come into contact with food allergens in utero. Hypotheses include the presence of CMP antigen in amniotic fluid or the passage of antigen via the placenta [8, 9, 11]. Edelbauer et al. support the latter hypothesis, identifying beta‐lactoglobulin and ovalbumin in both fetal and maternal blood sampled from placenta [12]. Additional indirect supportive evidence exists, demonstrating significant immune cellular proliferation and cytokine release in fetuses and neonates exposed to food allergens [13–15]. Moreover, Ward et al. [15] demonstrate elevated TNF‐alpha production by cord blood mononuclear cells exposed to common dietary proteins CMP and gliadin in a neonate presenting with food allergy and hypothesize this observation to be a result of intrauterine sensitization.

The development of food allergies is thus a complex process that could begin from the antenatal period as described by Warner et al. [16]. Genetic susceptibility, intrauterine environment, and postnatal events concerning hygiene, gut microbial colonization, and allergen exposure dictate allergy development. Interestingly, in our patient, family history was positive for early onset CMPA in a first cousin from the maternal side. At present, little is known on the genetic susceptibility for CMPA; however, associations with CMPA and certain genes and epigenetic changes have been observed in Dutch childhood populations [17, 18]. Therefore, current focus lies in better understanding such processes to discover preventative therapies and reduce the burden of CMPA [19, 20]. Future management will allow for better anticipation of neonatal complications and avoidance of potentially invasive investigations.

## 3. Conclusion

Our case is an extremely rare description of early onset of FPIAP due to CMPA in a neonate and demonstrates the importance of including this diagnosis as a possible cause of neonatal hematochezia in the first hours of life. We provide a step‐wise approach suited to the neonatal population, enabling physicians to conduct a thorough investigation while potentially avoiding unnecessary invasive and potentially harmful procedures. Finally, this case highlights current research interest in understanding not only the postnatal but also the antenatal and perinatal factors that may influence allergy development. Implications of these new findings on future preventative treatments for CMPA are important already from the neonatal period, earlier than previously believed, as suggested by this case report.

## Funding

The authors received no financial support for the research, authorship, and/or publication of this article. Open‐access publishing was facilitated by Universite de Lausanne, as part of the Wiley–Universite de Lausanne agreement via the Consortium of Swiss Academic Libraries.

## Consent

Written informed consent for patient information and images to be published was provided by a legally authorized representative.

## Conflicts of Interest

The authors declare no conflicts of interest.

## Data Availability

Data sharing is not applicable to this article as no datasets were generated or analyzed during the current study.
